# Spatial and Temporal Patterns of Endopolyploidy in Mosses

**DOI:** 10.3390/genes12010027

**Published:** 2020-12-27

**Authors:** Marianna Paľová, Dajana Ručová, Michal Goga, Vladislav Kolarčik

**Affiliations:** Institute of Biology and Ecology, Faculty of Science, P. J. Šafárik University, Mánesova 23, SK-041 54 Košice, Slovakia; palova.marianna6@gmail.com (M.P.); dajana.rucova@upjs.sk (D.R.); michal.goga@upjs.sk (M.G.)

**Keywords:** bryophytes, cycle value, endopolyploidy, flow cytometry, gametophyte, sporophyte

## Abstract

Somatic polyploidy or endopolyploidy is common in the plant kingdom; it ensures growth and allows adaptation to the environment. It is present in the majority of plant groups, including mosses. Endopolyploidy had only been previously studied in about 65 moss species, which represents less than 1% of known mosses. We analyzed 11 selected moss species to determine the spatial and temporal distribution of endopolyploidy using flow cytometry to identify patterns in ploidy levels among gametophytes and sporophytes. All of the studied mosses possessed cells with various ploidy levels in gametophytes, and four of six species investigated in sporophytic stage had endopolyploid sporophytes. The proportion of endopolyploid cells varied among organs, parts of gametophytes and sporophytes, and ontogenetic stages. Higher ploidy levels were seen in basal parts of gametophytes and sporophytes than in apical parts. Slight changes in ploidy levels were observed during ontogenesis in cultivated mosses; the youngest (apical) parts of thalli tend to have lower levels of endopolyploidy. Differences between parts of cauloid and phylloids of *Plagiomnium ellipticum* and *Polytrichum formosum* were also documented; proximal parts had higher levels of endopolyploidy than distal parts. Endopolyploidy is spatially and temporally differentiated in the gametophytes of endopolyploid mosses and follows a pattern similar to that seen in angiosperms.

## 1. Introduction

Endoreduplication (the type of endoreplication) is a DNA replication process in somatic cells that occurs in nuclei in which mitosis is skipped [1,2,3]. The result of this modified cell cycle, called the endocycle, is one nucleus with doubled DNA content [4,5]. The doubling of the amount of DNA in a cell can occur via multiple initiations of replication within one S phase, repetition of the S phase, or continuous alternation between S and G phase [6]. These cell cycle modifications are controlled by cyclins, cyclin-dependent kinases and various phytohormones, which positively or negatively regulate transitions to subsequent phases [7,8,9]. Endoreduplication leads to endopolyploidy (polysomaty, i.e., the presence of various levels of ploidy in cells within the plant tissue) [1]. Endopolyploidy is common in the plant kingdom [2,3], and it is quite common in plants with small genomes. Indeed, the size of a plants’ genome is negatively correlated with its eventual degree of endopolyploidization [1,6]. Endopolyploidization is present in several groups of algae and in mosses [10], although it is absent in lycophytes [10,11]. Ferns and cycads are not well studied in terms of endopolyploidy, but the phenomenon has been documented in a few fern species [8,10]. Endopolyploidy is particularly widespread in angiosperms, since it is assumed that more than 90% of herbaceous species show endopolyploidization [1,8]. It is well documented in the model species *Arabidopsis thaliana* (L.) Heynh. [12,13] and important crops, e.g., *Zea mays* L. [14,15] and *Sorghum bicolor* (L.) Moench [16]. However, there are plant groups, where endopolyploidy has not yet been documented; these include woody plants, gnetophytes, and other gymnosperms and some angiosperms [1]. Endopolyploidy probably evolved independently across multiple lines of plants [8,10]. Endopolyploidy is of great importance to plants. As the number of DNA copies in a cell increases, the cell elongates [14,17,18] and the cell wall is modified [12]. If the environmental conditions are unfavorable for cell division, plants can adapt using controlled transitions to the endocycle immediately following proliferation and thus ensure growth [8,18,19]. Endopolyploidization only takes place before tissues are fully developed [1].

Endopolyploidy is particularly advantageous for the growth of plants with small genomes [1,8]. According to the nucleotype theory, the size of the genome of an individual impacts its resulting phenotype, either due to the organism’s genotype or because of changes in cell size and the duration of each phase of the cell cycle [13]. Endopolyploidy is also beneficial in species that need to flower and produce seeds in the shortest time possible to avoid shading by other plants and in species that grow in frequently disturbed habitats [1]. In terms of cell differentiation, endopolyploidy is particularly important for specialized tissues that perform functions that require the supply of a higher number of metabolites [20]. Endoreduplication provides the necessary number of copies of DNA for given metabolites or additional organelles so that metabolic processes can proceed optimally [12,14,21]. The development of specific tissues, such as the endosperm of cereals and fruit pericarp, is often ensured by endopolyploidy [22].

Another important role of endopolyploidy in plants is its participation in response to stress caused by environmental stimuli [20]. Since plants are sessile organisms, they must employ special adaptive mechanisms to survive. Endoreduplication can help to alleviate oxidative stress by linking to the pentose cycle, where various secondary plant protection metabolites are formed [8]. Endopolyploidy is associated with abiotic stresses, including intense light [14,23,24], UV-B light [8,25,26], extreme temperature [8,27,28], water deficit [8,29,30], and high salinity [31,32,33,34]. Endoreduplication may be initiated by biotic stressors, phytohormones [4,8,21], allelopathy [8,35,36], or symbiosis [14,37]. An example of endoreduplication in symbiosis is colonization of roots by arbuscular mycorrhiza, which can lead to a higher level of endopolyploidy even in species that are not polysomatic [38]. Endoreduplication can mitigate apical meristem damage by herbivores [39,40,41]. It can also provide protection against stress caused by pathogens and insects by controlling the production of jasmonic acid [42]. Parasites can cause an increase in endopolyploidization in the roots of host plants, especially by secretion of cytokinins, which cause cells to switch from the cell cycle to the endocycle. This creates giant cells that eventually form galls that function as feeding sites for nematodes [43,44,45].

Most of our knowledge of endopolyploidy comes from the study of angiosperm models; however, endopolyploidy is very common in bryophytes, but unfortunately this plant group has attracted limited attention regarding endopolyploidy [10,36,46]. Bryophytes represent the second largest group of land plants in the world after angiosperms [47] and presently include three extant phyla: Bryophyta (mosses), Marchantiophyta (liverworts) and Anthocerotophyta (hornworts) [48]. Each of these three groups has a very long evolutionary history dating to more than 400 million years ago [49]. Mosses are pioneer organisms and are represented by ~13,000 species [50], while the liverworts include over 6000 species [47]. Mosses are widely distributed from the Arctic to Antarctica, and their ability to grow in many habitats contributes to their geographical distribution. They can inhabit a very wide range of ecosystems, habitats, microhabitats, and substrates that are unsuitable for vascular plants [51]. Nutrient-poor habitats are almost uninhabitable, but many species of mosses are nevertheless able to colonize them. Like other two bryophyte phyla, mosses have a gametophyte-dominated life cycle. In the photosynthetic phase of their life cycle, they exist as haploid gametophytes [52].

Liverworts and hornworts are sister groups of mosses. Despite their small genomes, endopolyploidy has not been shown to occur in these groups. In liverworts, besides 1C nuclei (monoploid containing one set of chromosomes), 2C nuclei (diploid containing two sets of chromosomes) were found in low frequency, but these may represent nuclei in G_2_ phase (of doubled DNA content entering mitosis) [53]. To date, no studied hornworts have been shown to have endopolyploid nuclei in their thalli [54]. In contrast, of the 65 moss species studied, almost all were endopolyploid [10,46]. Mostly, 1C to 4C nuclei were found, but some species had nuclei of up to 16C [10]. The exception was two species of the genus *Sphagnum* L. In mosses, a relationship between genome size and degree of endopolyploidization has not been confirmed, when analyzed in a phylogenetic context [10]. The stress response of some moss species includes alterations in growth rate and increase in the endopolyploidy level [36]. However, the previous study was not able to explain causal effects. Thus, it is possible that endopolyploidy level may change during moss growth and aging and in different parts of the thalli.

In the present study, we determined the spatial distribution of endopolyploidy in the thalli of selected moss species, either at the level of the whole plant body or at the level of particular organs (cauloids and phylloids). The temporal pattern of endopolyploidy distribution was further addressed in an in vitro experiment. With the new data obtained, it will be possible to analyze trends in endopolyploidy variability in mosses and compare them with known trends in angiosperms, resulting in improved understanding of the evolution and roles of this feature.

## 2. Materials and Methods

### 2.1. Plant Material

In the present study, investigated mosses were selected based on their availability (cultured plants), morphology (acrocarpous and pleurocarpous species) and size (small thallus, e.g., *Bryum moravicum* Podp., vs. large thallus, e.g., *Polytrichum formosum* Hedw.). Naturally growing mosses *Atrichum undulatum* (Hedw.) P. Beauv., *Brachythecium rivulare* Schimp., *B. moravicum*, *Dicranum scoparium* Hedw., *Eurhynchium striatum* (Hedw.) Schimp., *Plagiomnium ellipticum* (Brid.) T. J. Kop., *Plagiomnium undulatum* (Hedw.) T. J. Kop., *Polytrichastrum alpinum* (Hedw.) G.L. Smith, and *P. formosum* were screened for endopolyploidy. Collection data are given in summary Table 1.

*Physcomitrella patens* (Hedw.) Bruch and Schimp and *Pohlia drummondii* (Mül. Hal.) A. L. Andrews were used for analysis of changes in endopolyploidy in three ontogenetic stages. Both moss species were grown in aseptic culture on solid Benecke’s medium (composition: 200 mg·L^−1^ NH_4_NO_3_, 100 mg·L^−1^ MgSO_4_·7H_2_O, 400 mg·L^−1^ KH_2_PO_4_, and 100 mg·L^−1^ CaCl_2_·2H_2_O), which was solidified with 0.8% agar (VWR, Prolab) at a pH of 5.8 [55]. Cultivation took place under standard conditions: a temperature of 22 ± 2°C, 40% relative humidity, a 16 h/8 h (day/night) photoperiod, and an average artificial irradiance of 83.18 μM·m^−3^·s^−1^. Plant material was collected at three stages: after 14 (I stage), 26 (II stage), and 38 days (III stage).

### 2.2. Sample Preparation for Flow Cytometry and Measurements

**Figure 1:**
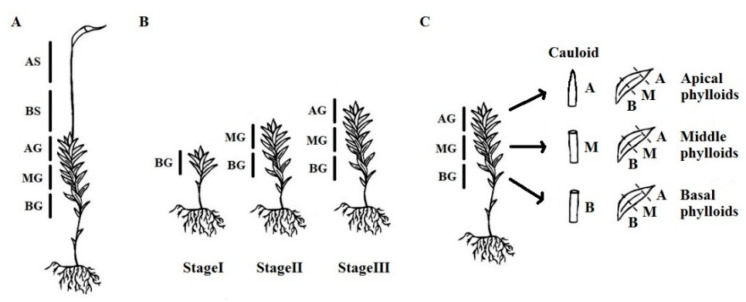
Schematic drawings of moss separation for sample preparation. (**A**) separation of gametophyte and sporophyte in Experiment 1, (**B**) separation of in vitro cultivated gametophytes in three ontogenetic stages, StageI, StageII and StageIII in Experiment 2, (**C**) separation of organs, cauloid and three spatially different types of phylloids in Experiment 3. BG, basal part of gametophyte; MG, middle part of gametophyte; AG, apical part of gametophyte; BS, basal part of sporophyte; AS, apical part of sporophyte; B, basal part of an organ; M, middle part of an organ; A, apical part of an organ.

In the study, five different individual thalli were collected from one up to five (depending on the species) moss cushions on the collection site. Therefore, they represent likely five replicates of the same individual (cultivated *P. patens* and *P. drummondii* or natural *B. moravicum*), but we cannot exclude the possibility that two or even five individuals were represented among five replicates (such as naturally growing mosses *P. alpinum* or *P. formosom*). Moss gametophyte thalli are differentiated into rhizoids (equivalent to roots), cauloids (stems), and phylloids (leaves). Moss sporophyte thallus is growing up from the gametophyte, and is differentiated into seta (capsule stalk), and capsule (sporangium). In the study, gametophytes (G, cauloid and phylloids were analyzed) were divided into three parts (BG—basal gametophyte, MG—middle gametophyte, and AG—apical gametophyte) and sporophytes (S, only seta was analyzed) were divided into two parts (BS—basal sporophyte and AS—apical sporophyte) (Figure 1A). Please note that sporophytes were only developed in *A. undulatum*, *B. rivulare*, *B. moravicum*, *D. scoparium*, *P. alpinum* and *P. formosum* in period of sampling. At least five replicates of each part of the thallus were prepared. In the cultivation experiment (Figure 1B), whole gametophytes of *P. patens* and *P. drummondii* were used in the first stage (Stage I, 14 days, approx. ~12 and ~18 phylloids were developed for *P. patens* and *P. drummondii*, respectively). In the second stage (Stage II, 26 days), gametophytes were divided into basal (~12 and ~18 phylloids in *P. patens* and *P. drummondii*, respectively) and apical parts (remainder of the thallus), and in the third stage (Stage III, 38 days), when they were of sufficient size, they were divided into three parts (~12 + ~12 + rest in *P. patens* and ~18 + ~18 + rest in *P. drummondii*). A total of five replicates were analyzed for both *P. patens* and *P. drummondii* in all three stages. To analyze spatial distribution of endopolyploidy in each part of organ, *P. formosum* and *P. ellipticum* plants were divided into the cauloid and phylloids. Phylloids were further collected from three parts of the gametophyte (BG, MG, AG). The cauloid (C) and phylloids (PBG, PMG and PAG) were divided into basal (B), middle (M), and apical parts (A) (Figure 1C). Three replicates were prepared for all combinations of factors (organ × part of organ) for both species.

Samples for flow cytometry (FCM) were prepared according to a standard method by chopping plant material in 1 mL of general purpose buffer (0.5 mmol·L^−1^ spermine. 4HCl, 30 mmol·L^−1^ sodium citrate, 20 mmol·L^−1^ MOPS, 80 mmol·L^−1^ KCl, 20 mmol·L^−1^ NaCl, and 0.5% [v/v] Triton X-100, pH 7.0) [56] in a Petri dish with a razor blade [56,57]. After filtering through 42 μm nylon filter, nuclei suspension was treated with RNAase (in final concentration of 30 μg·mL^−1^), propidium iodide (30 μg·mL^−1^), and with β-mercaptoethanol (2 μL·mL^−1^) as described in Goga et al. [36]. Samples were analyzed using a CyFlow ML cytometer (Partec GmbH, Münster, Germany). The resulting histograms were displayed on a logarithmic scale of relative fluorescence so that individual peaks were equally spaced [1,58]. The peaks were manually ranged and sized on FCM histograms as described [59] and represent the number of nuclei of respective ploidy levels. Since basic inherited ploidy levels alter between gametophyte and sporophyte, the correct nuclear ploidy level was determined either based on careful comparative inspection of both gametophyte and sporophyte (if this was available) or determination of genome size (in case of cultured plants). In case of plants represented just by the gametophyte (*P. undulatum*, *E. striatum*), there is a small risk of misinterpreting the 2C peak as the 1C peak on FCM records.

### 2.3. Calculation of Endoreduplication Index

Based on the number of nuclei of the individual peaks of the FCM histograms, the endoreduplication index (EI, syn. cycle value) of each gametophyte (reduced haploid phase) was calculated according to the following formula [1,60]:

EI = (0 × n_1C_ + 1 × n_2C_ + 2 × n_4C_ + 3 × n_8C_ + …)/(n_1C_ + n_2C_ + n_4C_ + n_8C_ + …).

Since the sporophyte represents an unreduced diploid phase, the EI was calculated according to the formula:

EI = (0 × n_2C_ + 1 × n_4C_ + 2 × n_8C_ + 3 × n_16C_ + …)/(n_2C_ + n_4C_ + n_8C_ + n_16C_ +…).

where n_1C,_ n_2C_, n_4C_, n_8C_, n_16C_… represents the number of nuclei of the respective ploidy levels. Tissues with an EI higher than 0.1 are considered endopolyploid [1].

### 2.4. Statistical Evaluation

Results were evaluated in the statistical software R, version 3.6.1 [61]. To test data normality, the Shapiro–Wilk test was applied. Homoscedasticity of data was confirmed by applying the Levene test. Outliers, which were rarely detected, were excluded from the dataset prior to statistical analyses. We used a one-way ANOVA (aov procedure in R) and Tukey’s HSD pairwise multiple comparison to test for differences between thalli parts. If data did not meet assumptions of ANOVA, then they were analyzed using the Kruskal–Wallis test with the Mann–Whitney test for pairwise multiple comparison with Benjamin and Hochberg correction. Furthermore, EI values were compared using a one-way ANOVA and t test to analyze temporal distribution of endopolyploidy in basal parts of the gametophyte (present in three stages) and in middle part of the gametophyte (present in two stages) in cultivation experiment (study of *P. patens* and *P. drummondii*). A two-way ANOVA test was used to analyze the spatial distribution of endopolyploidy in organs (study of *P. ellipticum* and *P. formosum*). Plots were visualized in ggplot2 ver. 2.2.1 [62].

## 3. Results

### 3.1. Spatial Distribution of Endopolyploidy in Moss Thalli

**Figure 2:**
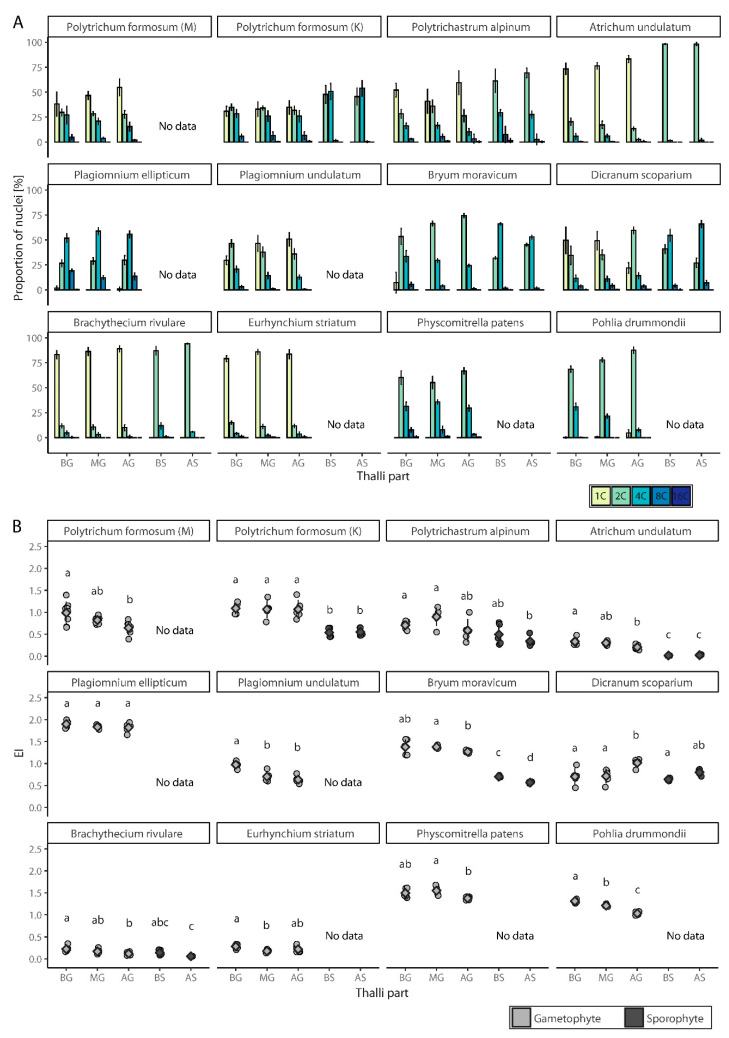
Endopolyploidy level in studied mosses. (**A**) Percentual distribution (mean ± SD) of different categories of nuclei. (**B**) Jitter plot with mean ± SD for endoreduplication index (EI) values. BG, basal part of gametophyte; MG, middle part of gametophyte; AG, apical part of gametophyte; BS, basal part of sporophyte; AS, apical part of sporophyte. Different populations of *P. formosum* are coded as M and K, reflecting name of collection site; M, Milpoš; K, Kamenica. Homogenic groups revealed by ANOVA (or Kruskal-Wallis test) and Tukey’s HSD test (or Mann-Whitney post hoc test) are denoted by lower case letters.

**Figure 3:**
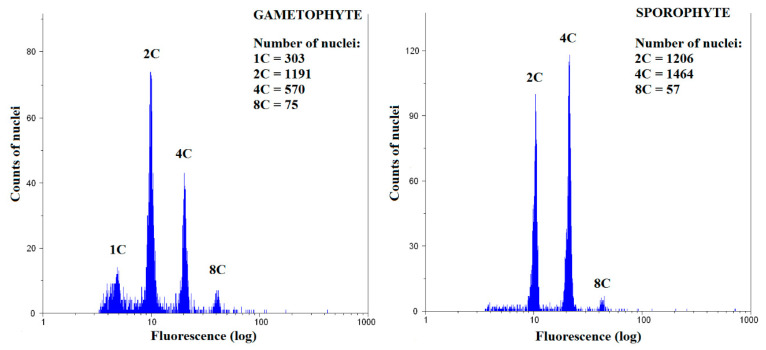
Representative flow cytometry histogram for *Bryum* *moravicum* gametophyte and sporophyte.

**Figure 4:**
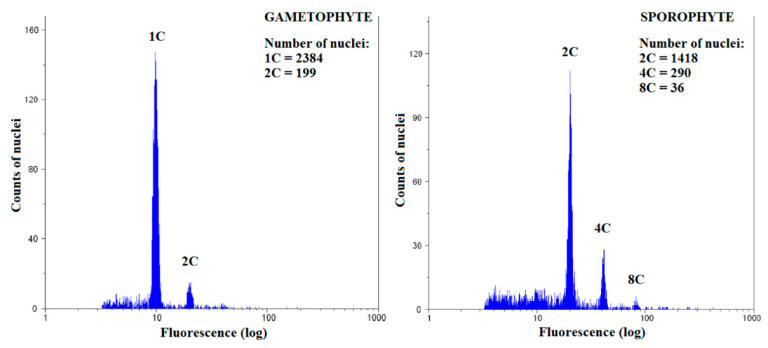
Representative flow cytometry histogram for *Brachythecium rivulare* gametophyte and sporophyte.

To study the spatial distribution of endopolyploidy, nine naturally growing and two laboratory-cultured moss species were analyzed: *Atrichum undulatum*, *Brachythecium rivulare*, *Bryum moravicum*, *Dicranum scoparium*, *Eurhynchium striatum*, *Physcomitrella patens* (laboratory culture), *Plagiomnium ellipticum*, *Plagiomnium undulatum*, *Pohlia drummondii* (laboratory culture), *Polytrichastrum alpinum*, and *Polytrichum formosum*. All the studied mosses had 1C, 2C, and 4C nuclei present in the gametophyte, except P. patens (Figure 2A, Supplementary Table S1). In addition, 8C peaks were observed in the basal, middle, and apical parts of the gametophytes of all mosses, except *B. rivulare* and *P. drummondii*. A portion of nuclei in *P. formosum*, *P. alpinum*, *P. ellipticum*, *D. scoparium*, and *P. patens* had ploidy level of 16C at least in some of their samples. The lowest endoreduplication index (EI) values for gametophytes were recorded in *B. rivulare* (AG, EI = 0.12 ± 0.04), while the highest values were recorded in *P. ellipticum* (BG, EI = 1.90 ± 0.08) (Figure 2B, Supplementary Table S1). It is worth mentioning that *P. ellipticum*, *B. moravicum*, *P. patens*, and *P. drummondii* contained very low or no 1C nuclei, and dominant and often the first peak in gametophytes was identified as a 2C peak (compare Figure 3 and Figure 4).

In the time of analysis, only six moss species (*A. undulatum*, *B. rivulare*, *B. moravicum*, *D. scoparium*, *P. alpinum* and *P. formosum*) had developed sporophytes. In all cases, sporophytes had lower levels of endopolyploidy compared to gametophytes. Mostly 2C, 4C and 8C nuclei were represented in the analyzed sporophytes, with 8C nuclei absent only in the sporophytes of *A. undulatum* (Figure 2A, Supplementary Table S1). Up to 16C nuclei were present in some samples of sporophytes of *B. rivulare*, *B. moravicum*, *D. scoparium*, and *P. alpinum*. The cells of the basal sporophyte of *B. rivulare* rarely reached 16C levels, and the cells of the apical sporophyte were much lower, only of 2C and 4C, and EI did not exceed 0.1; therefore it is not considered to be endopolyploid. The later was observed also for sporophyte of *A. undulatum*. The EI varied from the lowest values found in *A. undulatum* (BS, 0.02 ± 0.01, non-endopolyploid) to the highest values in *D. scoparium* (AS, 0.80 ± 0.06) (Figure 2B, Supplementary Table S1).

Differences in endopolyploidy levels (expressed as EI values) were statistically significant between gametophytic and sporophytic tissues (whichever parts are considered) in *P. formosum*, *A. undulatum* and *B. moravicum*, and EI values were lower in sporophytes compared to gametophytes in these cases (Figure 2B).

In gametophytes, we recorded significantly different EI values among various parts of the gametophyte (BG, MG, and AG) in all the species investigated except *P. ellipticum* and *P. formosum*, the later collected from locality near Kamenica (Figure 2B). In contrast, both investigated sporophytic parts (BS and AS) did not differ in all the species analyzed except *B. moravicum*; and in this case, EI values were higher in the BS compared to the AS. All the investigated species tended to have lower EI values in apical parts except *D. scoparium*, which showed the opposite pattern.

To generalize our results, two within-tissue ploidy level distribution patterns were observed. Some species, such as *B. rivulare*, *E. striatum*, *A. undulatum* or *P. drummondii*, had cells of one dominant ploidy level; this could be 1C or 2C in gametophyte or 2C in sporophyte. Other endopolyploid cells were found often only at quite low levels. Other species, including *P. formosum*, *P. unudulatum*, *D. scoparium* or *P. patens*, showed a pattern of almost continuous increase or decrease of nuclei counts in subsequent ploidy classes. This resulted into almost equal proportions of two or three nuclei classes, as in case of *P. formosum*.

Interestingly, we have found variable EI pattern in *P. formosum* represented here by two spatially and timely different samples. While individuals lacking sporophytes collected in Milpoš (in 2018) were variable in three gametophytic parts, those with sporophytes and collected in Kamenica (in 2020) were not. This result points to the possibility that site or seasonal differentiation, genotype or population differentiation, or possibly sexual differentiation, may shape overall plant endopolyploidy level.

### 3.2. Spatiotemporal Analysis of Endopolyploidy in Two Moss Species Cultivated In Vitro

To analyze changes in endopolyploidy in relation to organ or tissue age, *P. patens* and *P. drummondii* were grown under laboratory conditions and were compared at three stages of development. In *P. patens*, 8C nuclei were observed in all parts of the moss (and stages) except for the apical portion developed in the third stage. In *P. drummondii*, 8C nuclei were recorded only in first stage of plants and the basal part of plants in both the second and third stages. In the other samples, the highest ploidy level was 4C. Generally, *P. patens* showed slightly higher EI values.

#### 3.2.1. *Physcomitrella Patens*

**Figure 5:**
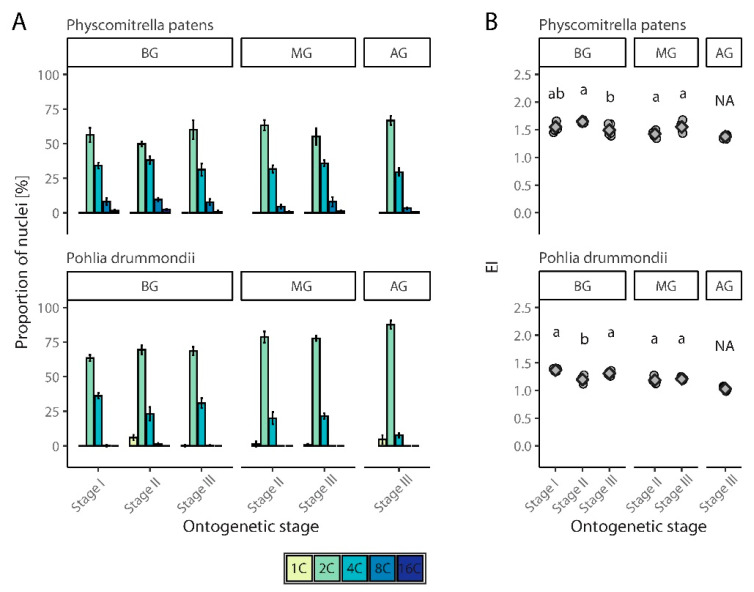
Spatial and temporal endopolyploidy pattern in cultivated moss species, *Physcomitrella patens* and *Pohlia drummondii*. (**A**) Percentual distribution (mean ± SD) of different categories of nuclei of studied mosses gametophyte in three ontogenetic stages. (**B**) Jitter plot with mean ± SD for endoreduplication index (EI) values of studied moss species. BG, basal part of gametophyte; MG, middle part of gametophyte; AG, apical part of gametophyte. Homogenic groups revealed by ANOVA (or t test in case of MG) and Tukey’s HSD test are denoted by lower case letters. NA, not applied.

The proportion of 1C nuclei was high in *P. patens* in the first stage of analysis, and 2C nuclei were present in smaller proportions. Only a few dozen of 4C nuclei were detected (Figure 5A, Supplementary Table S1). Similarly, all four peaks were detected in almost the same proportions in the second stage. The variance in the percentage of nuclei of different categories was more evident in the third stage of the analysis. The presence of 2C and 4C nuclei also gradually decreased in the third stage. 8C nuclei were observed in the basal and middle parts of the gametophyte, but were completely absent in the apical region of the gametophyte (Figure 5A). Statistical tests showed that the differences between EI, according to this ontogenetic stage of tissue, were significant compared to the basal part of the gametophyte (ANOVA, F(2, 12) = 4.39, *p* < 0.05) but not for middle part of gametophyte (*t* test, t = −2.05, df = 4.35, *p* > 0.05) (Figure 5B, Supplementary Table S1).

#### 3.2.2. *Pohlia Drummondii*

In the first stage of cultivation, three to four peaks were present on the FCM histograms, representing nuclei with ploidy levels of 1C, 2C, 4C, and 8C. The most frequent ploidy level was 2C; 1C cells were missing completely in some replicates or were present only in relatively small numbers (Figure 5A, Supplementary Table S1). In the second stage of cultivation, 8C nuclei were not observed, and the highest ploidy level was 4C. Similarly, at this stage, most tissues consisted of 2C cells, and the number of 1C cells decreased compared to the first stage. On the other hand, an increase in the number of 4C cells was observed (Figure 5A). In the third stage, the majority of cells in the gametophyte were 2C. In the middle part of the gametophyte, there were only a few 1C nuclei; meanwhile, in the apical part, their numbers increased. The highest level of endopolyploidy for both these parts of the gametophyte was 4C, although several 8C nuclei were detected in the basal portion (Figure 5A). Statistical tests confirmed the significance of differences between EI values recorded for different ontogenetic stages for the basal (ANOVA, F(2, 12) = 19.96, *p* < 0.001), but not for middle part of gametophyte (*t* test, t = −0.82, df = 5.73, *p* > 0.05) (Figure 5B, Supplementary Table S1).

### 3.3. Spatial Distribution of Endopolyploidy in Moss Organs

Two mosses, *P. formosum* and *P. ellipticum*, were used to analyze the distribution of endopolyploidy within the cauloids and phylloids originating from different parts of the gametophyte. Both species had relatively high levels of endopolyploidization, with 1C to 8C nuclei present in their gametophytes. In the gametophytes of *P. ellipticum*, 16C cells were also represented in the basal and middle parts of the cauloids. In general, the degree of endopolyploidization in the cauloid and phylloids of both species decreased from base to apex. *Plagiomnium ellipticum* had a higher endopolyploidy level in both the cauloids and phylloids compared to *P. formosum.*

#### 3.3.1. *Polytrichum Formosum*

**Figure 6:**
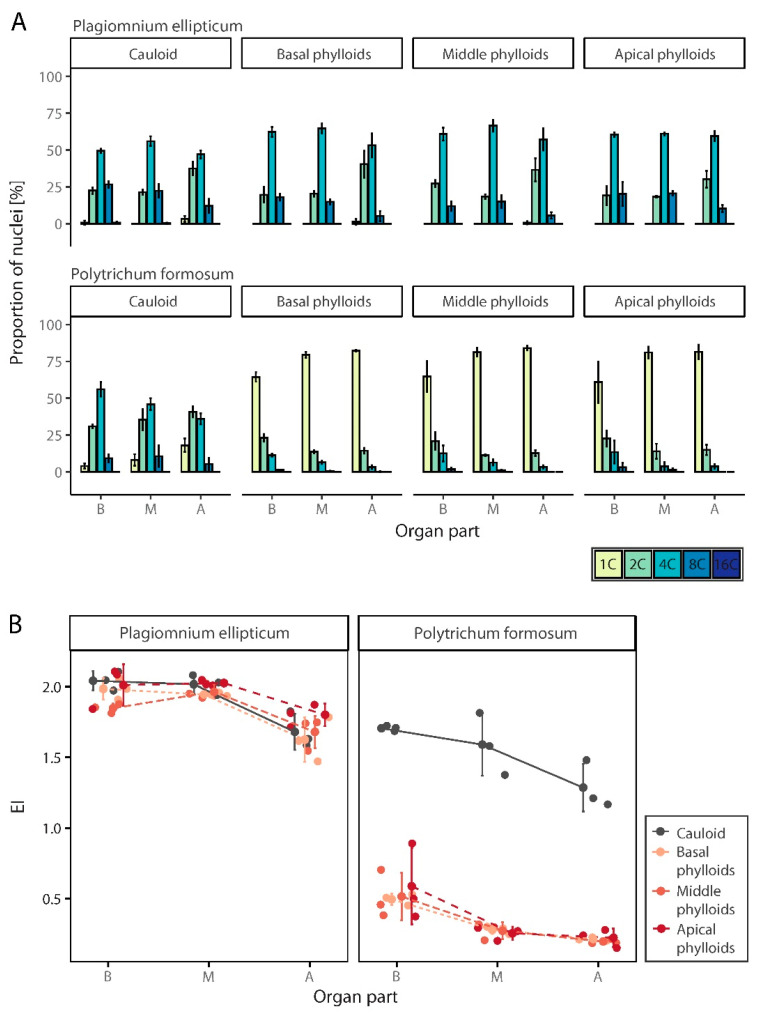
Spatial endopolyploidy pattern in naturally growing moss species, *Plagiomnium ellipticum* and *Polytrichum formosum*. (**A**) Percentual distribution (mean ± SD) of different categories of nuclei isolated from separated organ and organ parts of two moss species. (**B**) Jitter plot with mean ± SD for endoreduplication index (EI) values of studied moss species, their organs and organ parts. B, basal part of an organ; M, middle part of an organ; A, apical part of an organ.

In all three parts of gametophytes of P. formosum, 1C to 8C cells were represented irrespective of organ, cauloid or phylloids. These four cell categories were also present in the analyzed parts of all phylloids, except in the apical portion of the middle and apical phylloids of the gametophytes. Only three peaks were observed in these sections; the fourth peak, representing 8C nuclei, was absent. Most of the basal parts of the cauloids were composed of 4C nuclei; 2C and 8C nuclei were present in smaller amounts, and 1C cells were least abundant. 4C cells again dominated in the middle part of the cauloid, but the number of 1C nuclei dominated in the cauloid base (Figure 6A, Supplementary Table S1).

In phylloids, the 1C nuclei were most abundant, regardless of which part of the moss from which the phylloid was sampled. Within individual phylloids, a trend of decreasing ploidy level from base to apex was observed. While the number of 1C nuclei increased, there was a decrease in the number of 8C nuclei (Figure 6A), which were almost absent in the apical portion of the phylloids. This was reflected by a decrease in EI (Figure 6B, Supplementary Table S1). Differences between organs (cauloids, three types of phylloids) (two-way ANOVA, F(3, 24) = 200.0; *p* < 0.001) and among organ parts (basal, medium, apical) (F(2, 24) = 23.3; *p* < 0.001) were statisticaly significant, but interaction between them was not (F(6, 24) = 0.8; *p* > 0.05). A Tukey post hoc test revealed significant pairwise differences between cauloid and all three types of phylloids, showing 1.17 up to 1.20 higher EI values, and between the basal and both the middle (- 0.23 EI) and apical parts of organs (- 0.35 EI).

#### 3.3.2. *Plagiomnium Ellipticum*

In *P. ellipticum*, five peaks in cauloids and three to four peaks in phylloids were detected by FCM. In addition to 1C, 2C, 4C, and 8C nuclei, 16C nuclei were observed throughout the cauloid except in the apical part (Figure 6A, Supplementary Table S1). In the phylloids taken from the basal and middle parts of the organs, 2C, 4C, and 8C nuclei dominated. Cells at the baseline level of 1C were again minimally present and were found only in the apical parts of phylloids growing at the base and in the middle of the gametophyte. In phylloids removed from the apical parts of the moss, 1C cells were completely absent (Figure 6A).

This species had relatively high EI values and similar pattern of variation for both cauloid and phylloids. Within these organs, endopolyploidy decreased from the basal and middle parts to the apical parts (Figure 6B, Supplementary Table S1). Differences between organs (cauloids, three types of phylloids) (two-way ANOVA, F(3, 24) = 3.0; *p* > 0.05) were not statisticaly significant, while differences among organ parts (basal, middle, apical) (F(2, 24) = 38.1; *p* < 0.001) were. Interaction between them was not statisticaly significant (F(6, 24) = 1.0; *p* > 0.05). A Tukey post hoc test revealed significant pairwise differences between the apical part of organs and both basal and middle portions (showing 0.27 and 0.29 lower EI values compared to basal and middle parts, respectively).

## 4. Discussion

Endopolyploidy, or increased ploidy in somatic cells beyond inherited organism ploidy level, is widespread in some plant groups, e.g., mosses and angiosperms, and has been reported to have various effects from the cellular to whole organism level. Recent studies have primarily focused on the study of endopolyploidy in higher plants or the model moss *Physcomitrella patens* [36,63]. To date, studies on mosses focused on investigation of evolutionary patterns, and moss thalli were analyzed as a whole. Detailed data about endopolyploidy variation across different tissues of moss thalli are missing (but reported to be well known by Bainard et al. [46]). Therefore, in the present study, we analyzed the distribution of endopolyploidy, in selected moss species, in different parts of thalli, at various ontogenetic stages in individual plants and the spatial abundance of endopolyploid cells within different organs of these plants.

### 4.1. Spatial Distribution of Endopolyploidy in Moss Thalli and Organs

Levels of endopolyploidy vary depending on different factors, including the type of tissue or organ [1,6]. In angiosperms, differences between organs positioned lower and higher on the stem were previously reported [1]. Such differences are thought to be related to differences in the formation of these organs during ontogenesis. To the best of our knowledge, this topic has not been explored in mosses so far. Any study addressing endopolyploidy variation in mosses assumed homogenic distribution of cells with various ploidy levels in thalli or ignored possible variation at all. The present study is the first to attempt to shed light on this issue. We identified a very similar tendency in mosses to that seen in angiosperms. Various levels of endopolyploidy were observed among the different moss species between gametophytes and sporophytes and also between each part of the gametophytes and sporophytes. Usually, older parts have a higher endopolyploidy level than younger ones. Our experiments suggest that older basal parts show increased endopolyploidy from their initiation and that the endopolyploidy level of tissue does not increase with age.

Gametophytes are established in the life cycle of mosses before sporophytes [64]; therefore, the gametophyte is ontogenetically older. In angiosperms, the level of endopolyploidization has been documented to be higher in older parts of plants [1,33,65]. The gametophytes of analyzed species generally had more endopolyploid cells than sporophytes, but the two parts were different in terms of basic (“inherited”) ploidy level. Therefore, lower endopolyploidy level documented in sporophytes, when compared to gametophytes, cannot be interpreted as a results of ontogenetically different, younger, origin of sporophytes. Variance in ploidy levels between gametophytes and sporophytes results from differences in the basic condition of nuclei, which is haploid and diploid, respectively. It is possible that higher endopolyploidy in gametophytes (and in mosses overall) could appear in evolution as compensation for haploidy [66]. Several copies of genes with cumulative effects on the eventual phenotype lead to a higher number of products of a given gene and therefore to faster synthesis of proteins. This can contribute to faster growth, development, and metabolic processes [8,12,20], allowing the gametophyte to produce sufficient metabolites for nourishment of the sporophyte. Alternatively, endopolyploidy may simply compensate for low DNA content in gametophytic (haploid) or sporophytic (diploid) tissues and help to promote growth and development through nucleotypic effects. Recent studies of endopolyploidy in diploid and isogenic polyploid angiosperms have revealed that diploids tend to show higher endopolyploidy than autopolyploids [41,67], possibly due to compensation for lower basic inherited organism-specific ploidy. These studies, however, conclude that this pattern is not general but genotype-specific.

Divergences in eventual endopolyploidy levels were observed in individual organs and parts of sporophytes and gametophytes in the present study. Endopolyploidization in apical parts of gametophytes and sporophytes was lower than in basal parts and tended to decrease along these organs from base to apex. Although the effect of interaction between factors on endopolyploidy in different parts of moss organs (organ vs. part of an organ) was investigated in the present study, the results cannot be generalized yet. This topic was investigated in two different species, *Plagiomnium ellipticum* and *Polytrichum formosum*. While the part of the moss thallus from which organs were removed was always significant, the differentiation in EI between organs (cauloid vs. spatially different phylloids) was documented only in *P. formosum*. The variability of endopolyploidization is specific to each species. For instance, in *P. formosum* we have observed that the cauloid has much higher EI values compared to the phylloids from any location on the gametophyte. In contrast, *P. ellipticum* did not show this pattern of differentiation. It is necessary to analyze more moss species to generalize trends in endopolyploidy patterns, particularly in organs and their positions on moss thalli. Cytohistological techniques coupled with confocal microscopy may be beneficial.

*Physcomitrella patens*, *P. drummondii*, *B*. *moravicum*, and *P. ellipticum* were characterized by replication of the whole genome in most cells in the gametophytes in the present study. In several samples, the first 1C peak was represented by very few or no nuclei, which may results into misinterpretation of FCM records [36,46]. In the case of *B*. *moravicum*, which had developed sporophytes at the time of analysis, the dominant and often the first peak in gametophytes was identified as a 2C peak. This suggests that 1C cells may often be absent in the gametophytes of some moss species. This is very interesting because it suggests that almost all gametophyte cells may harbor a completely reduplicated genome. However, here we consider an alternative explanation of reduplicated genome. Endopolyploidy in mosses is poorly explored so far. Most data come from studies on the model moss *P. patens*. This species is characterized by a specific, tissue-dependent cell cycle. The protonema of this moss consists of two types of tissues, caulonema and chloronema, but only the latter form in young individuals. Most chloronema cells undergo DNA synthesis during the S phase of the cell cycle early after mitosis and are usually found “timely arrested” just before transition from G_2_ phase to M phase. Therefore, screening for nuclei ploidy levels in this moss revealed mostly 2C nuclei [63]. Gradually, after the formation of caulonema, in which cells are mainly in the transition between G_1_ and the S phase, the proportion of 1C nuclei is shown to increase in tissue FCM cytotyping. Later, as the cells of the caulonema age, DNA endoreduplication can be detected [63], confirming that higher levels of endopolyploidy are found in older tissues [1,6]. However, there is no way to differentiate between the two routes of 2C nuclei origin (endopolyploidization or arrest of most cells in the G_2_/M phase). Indeed, Bainard et al. [46] stressed that such specific G_2_/M arrest of the cell cycle may lead to misinterpretation of FCM data in genome size estimation. Although Bainard et al. [46] mentioned that laboratory-cultured plants exhibited this type of cell cycle, to our knowledge, there have been no study published that exclude the possibility that even mosses growing naturally contain cells arrested in the G_2_/M phase. More detailed, future studies are needed to investigate this phenomenon in relation to endopolyploidy of mosses and further if the G_2_/M phase arrest of cells is ploidy-dependent.

### 4.2. Temporal Distribution of Endopolyploidy in Moss Thalli

As in various organs and tissues, endopolyploidy changes during ontogenesis in an individual. Endoreduplication in cells can continue until maturation [1] and therefore some differences occur within a type of tissue depending on the age of the organ. In angiosperms, ontogenetically older organs and tissues generally show higher degrees of endopolyploidy than younger ones [33,65,68], with some exceptions. For instance, decreased endopolyploidy in older roots and hypocotyls compared to their younger counterparts was observed in *Chenopodium quinoa* Willd. [69], *Trifolium pratense* L. [68], and *Beta vulgaris* L. [17]. 

This phenomenon occurs not only in higher plants, but also in mosses, as documented in the present study. As exemplified by *P. drummondii* and *P. patens*, a decrease in endopolyploidy from the base to the apex of gametophytes was demonstrated. In both species, higher ploidy levels were recorded in ontogenetically older parts of the thalli. The same patterns have been observed in higher plants [30,57]. A higher level of endopolyploidy found in the basal part (or middle part in *P. patens*) of gametophytes may be alternatively the result of faster thalli growth via increasing cell division leading to the detection of a higher proportion of cells entering the M phase compared to other stages. The observed inconsistent patterns of endopolyploidy variation in the part of a gametophyte during ontogenesis found in two selected species invites further in-depth analysis of ontogenetic patterns in several mosses, preferably those with a large thallus.

## 5. Conclusions

There were statistically significant differences in the levels of endopolyploidy between different parts of gametophytes and sporophytes in the studied mosses. In general, we observed higher degrees of endopolyploidization in gametophytes than in sporophytes. Based on the present study of 11 moss species, the level of endopolyploidy tends to be higher in basal parts of mosses than in apical parts. Also, endopolyploidy tends to decrease from basal to apical parts of the cauloids and phylloids. In addition, during analysis of endopolyploidy in selected moss species, it was found that some species replicated nearly all (or all) of the nuclear DNA content in gametophyte cells (*B*. *moravicum*, *P. patens*, *P. ellipticum*, and *P. drummondii*). Nuclei that were in the baseline 1C state were of minimal proportions compared to those in the endopolyploid states.

Research on endopolyploidy has so far been directed toward higher plants, although endopolyploidy is present in several moss species. As such, this group of plants is promising in terms of research on endopolyploidy as a part of adaptation to stress in plants or on the evolutionary patterns of endopolyploidy. Recent reports that endopolyploidy is common in mosses and in moss thalli and organs ([46], present study) provide opportunities to design research experiments with new perspectives and increased precision.

The present study shows that certain cytological features associated with thalli development in mosses have just started to be explored, and that further elucidation of these features may help to better understand moss biology and evolution. Mosses are resilient and ubiquitous organisms and play an important role in several ecosystem processes, including net primary productivity, decomposition rates, above-ground microclimate modifications, and carbon and nutrient cycling [70]. Bryophytes are important for global exploration of life as they are sister to vascular plants and are the extant lineage most similar to the first established land plants [71]. Consequently, their study may help to understand land colonization.

## Figures and Tables

**Table 1:** Summary of plant material used in the present study.

Species	Collection Details	Sample Size		
		Exp1	Exp2	Exp3
		G/S	StI/StII/StIII	C/PBG/PMG/PAG
Naturally growing plants				
*Atrichum undulatum*	ES, Milpoš, 49.193448° N, 21.015777° E	5/5	-/-/-	-/-/-/-
*Brachythecium rivulare*	ES, Milpoš, 49.193448° N, 21.015777° E	5/5	-/-/-	-/-/-/-
*Bryum moravicum*	ES, Milpoš, 49.193448° N, 21.015777° E	5/5	-/-/-	-/-/-/-
*Dicranum scoparium*	ES, Vysoké Tatry, 49.211943° N, 20.308818° E	5/5	-/-/-	-/-/-/-
*Eurhynchium striatum*	CS, Stankovany, 49.154203° N, 19.151767° E	5/-	-/-/-	-/-/-/-
*Plagiomnium ellipticum*	ES, Drahňov, 48.569892° N, 21.955160° E	5/-	-/-/-	3/3/3/3
*Plagiomnium undulatum*	ES, Rovné, 49.2736989° N, 21.5209615° E	5/-	-/-/-	-/-/-/-
*Polytrichastrum alpinum*	ES, Vysoké Tatry, 49.151811° N, 20.080580° E	5/5	-/-/-	-/-/-/-
*Polytrichum formosum*	ES, Milpoš, 49.193448° N, 21.015777° E (M code)	8/-	-/-/-	3/3/3/3
	ES, Kamenica, 49.212205° N, 20.991363° E (K code)	5/5	-/-/-	-/-/-/-
Laboratory culture				
*Physcomitrella patens*	-	5/-	5/5/5	-/-/-/-
*Pohlia drummondii*	-	5/-	5/5/5	-/-/-/-

List of taxa studied (Species) are characterized (Collection data) with locality, latitude, and longitude. Different populations of *P. formosum* are coded as M and K, reflecting name of collection site; M, Milpoš; K, Kamenica. Number of replicates analyzed in various experiments (Exp1–Exp3). Different parts of thallus, gametophyte (G) and sporophyte (S), different organs of gametophyte, cauloid (C), basal phylloids (PBG), middle phylloids (PMG) and apical phylloids (PAG) were analyzed as well as moss thalli of selected species at three ontogenetic stages (StI–StIII). ES and CS denote Eastern Slovakia and Central Slovakia respectively.

## Data Availability

All data generated or analyzed during this study are available from the corresponding author on reasonable request.

## Supplementary Materials

The following are available online at https://www.mdpi.com/2073-4425/12/1/27/s1, Table S1: Summary of exploratory statistics.
